# Overexpressing *TPTE2* (*TPIP*), a homolog of the human tumor suppressor gene *PTEN*, rescues the abnormal phenotype of the *PTEN^−/−^* mutant

**DOI:** 10.18632/oncotarget.24941

**Published:** 2018-04-20

**Authors:** Daniel F. Lusche, Emma C. Buchele, Kanoe B. Russell, Benjamin A. Soll, Michele I. Vitolo, Michael R. Klemme, Deborah J. Wessels, David R. Soll

**Affiliations:** ^1^ Developmental Studies Hybridoma Bank and W.M. Keck Dynamic Image Analysis Facility, Department of Biology, The University of Iowa, Iowa City, 52242 IA, USA; ^2^ Greenebaum Cancer Center, The University of Maryland, Baltimore, Maryland, Baltimore, 21201 MD, USA

**Keywords:** cancer cell rescue, tumor suppressor, transmembrane phosphatase, TPTE2 TPIP, wound healing

## Abstract

One possible approach to normalize mutant cells that are metastatic and tumorigenic, is to upregulate a functionally similar homolog of the mutated gene. Here we have explored this hypothesis by generating an overexpressor of *TPTE2* (*TPIP*), a homolog of *PTEN*, in *PTEN^−/−^* mutants, the latter generated by targeted mutagenesis of a human epithelial cell line. Overexpression of *TPTE2* normalized phenotypic changes associated with the PTEN mutation. The *PTEN^−/−^*-associated changes rescued by overexpressing *TPTE2* included 1) accelerated wound healing in the presence or absence of added growth factors (GFs), 2) increased division rates on a 2D substrate in the presence of GFs, 3) adhesion and viability on a 2D substrate in the absence of GFs, 4) viability in a 3D Matrigel model in the absence of GFs and substrate adhesion 5) loss of apoptosis-associated annexin V cell surface binding sites. The results justify further exploration into the possibility that upregulating *TPTE2* by a drug may reverse metastatic and tumorigenic phenotypes mediated in part by a mutation in *PTEN*. This strategy may also be applicable to other tumorigenic mutations in which a homolog to the mutated gene is present and can substitute functionally.

## INTRODUCTION

Loss of function of tumor suppressor genes, like *PTEN*, can facilitate tumorigenesis and metastasis, and in nontumorigenic cell lines, can result in phenotypic changes associated with both processes [1–12]. One way to rescue loss of function of a tumor suppressor gene, is to reintroduce the normal gene, in an expression plasmid into the cells [4, 8, 13–20]. This approach shows promise, but to date has not yielded effective treatments [21, 22]. There is, however, an alternative and, possibly, less intrusive strategy that has not been explored. If the defective gene contributing to tumorigenesis has one or more functional homologs, then stimulating overexpression of one of them in mutant cells may restore the function of the defective gene. *PTEN* indeed has two homologs, *TPTE1* and *TPTE2* (*TPIP*), a *PTEN* pseudogene [23–27], a *TPTE1* pseudogene [23] and seven *TPTE2* (*TPIP*) pseudogenes [23–28] (www.ncbi.nim.nih.gov/gene; www.uniprot.org Q6XPS3; www.ensemble.org). By over-expressing a homolog of *PTEN* with similar catalytic and membrane-binding domains in a cell that has lost PTEN function such as *TPTE2* (*TPIP*), one may be able to normalize the aberrant phenotype caused by the absence of *PTEN*. And, if expression of the homolog could be up-regulated by a surface receptor, and a chemical or monoclonal antibody could be identified that activates that receptor, one might be able to design a drug that reestablishes PTEN function in *PTEN* mutants and thus suppresses tumorigenesis and metastasis.

Recently, we explored this hypothesis by testing it in a model system, the simple amoeba *Dictyostelium discoideum*, which has been used for over 65 years as an exceptional model for human development and white blood cell function [29]. *D. discoideum* contains an ortholog of the human *PTEN* gene, *ptenA*, and a homolog of *ptenA*, *lpten*. Both PtenA and Lpten contain the two signature domains of human PTEN, the CDC14-protein tyrosine phosphatase domain and the lipid C2-binding domain [30]. The *ptenA* null mutant, *ptenA^−^*, exhibits strong behavioral defects in motility, chemotaxis, aggregation and multicellular morphogenesis [31–37]. The null mutant of the homolog, *lpten^−^*, behaves as a very weak phenocopy of *ptenA^−^* [30]. Overexpressing full length *lpten* rescued all developmental and behavioral defects of the *ptenA*^−^ mutant [30], an observation that provided initial support for the proposed hypothesis.

Here, we have used a similar strategy to test whether overexpressing *TPTE2*, also known as a homolog of *PTEN*, in a human *PTEN* null mutant, *PTEN^−/−^*, generated in the nontumorigenic breast epithelial cell line, MCF-10A [10], reversed characteristics associated with the loss of PTEN function –i.e., rescued the mutant *PTEN^−/−^* phenotype. The *PTEN^−/−^* strain was generated by targeted homologous recombination, and previously demonstrated to differ from the parent strain MCF-10A in a number of phenotypic characteristics [9, 10]. The *PTEN^−/−^* -associated phenotypes explored here include 1) an increased rate of wound healing, accentuated by the removal of growth factors (GFs) from the supporting medium, 2) an increase in the rate of cell division in the presence of GFs, 3) adherence and viability on a 2D substrate in the absence of GFs, 4) viability in a 3D Matrigel model in the absence of GFs and substrate adhesion, and 5) loss of the apoptosis-associated characteristic of annexin V binding and endocytosis [38–42]. We show that overexpressing *TPTE2* (*TPIP*) in *PTEN*^−/−^ mutant cells reverses all of the phenotypic changes associated with the *PTEN* mutation and in the cases of wound healing and annexin V binding, actually accentuates the normal *PTEN^+/+^* phenotype. These results add further support to the hypothesis that a receptor mediated signal that upregulates a homolog of a defective tumor suppressing gene can normalize defects in mutant cells associated with the mutation, with the potential of suppressing tumorigenesis and metastasis and stimulating apoptosis. This approach may also prove effective for other types of cancer-associated mutations, for which the mutant gene has a functional homolog that can be upregulated.

## RESULTS

### Generating *PTEN^−/−^TPTE2^OE^* strains

**Figure 1:**
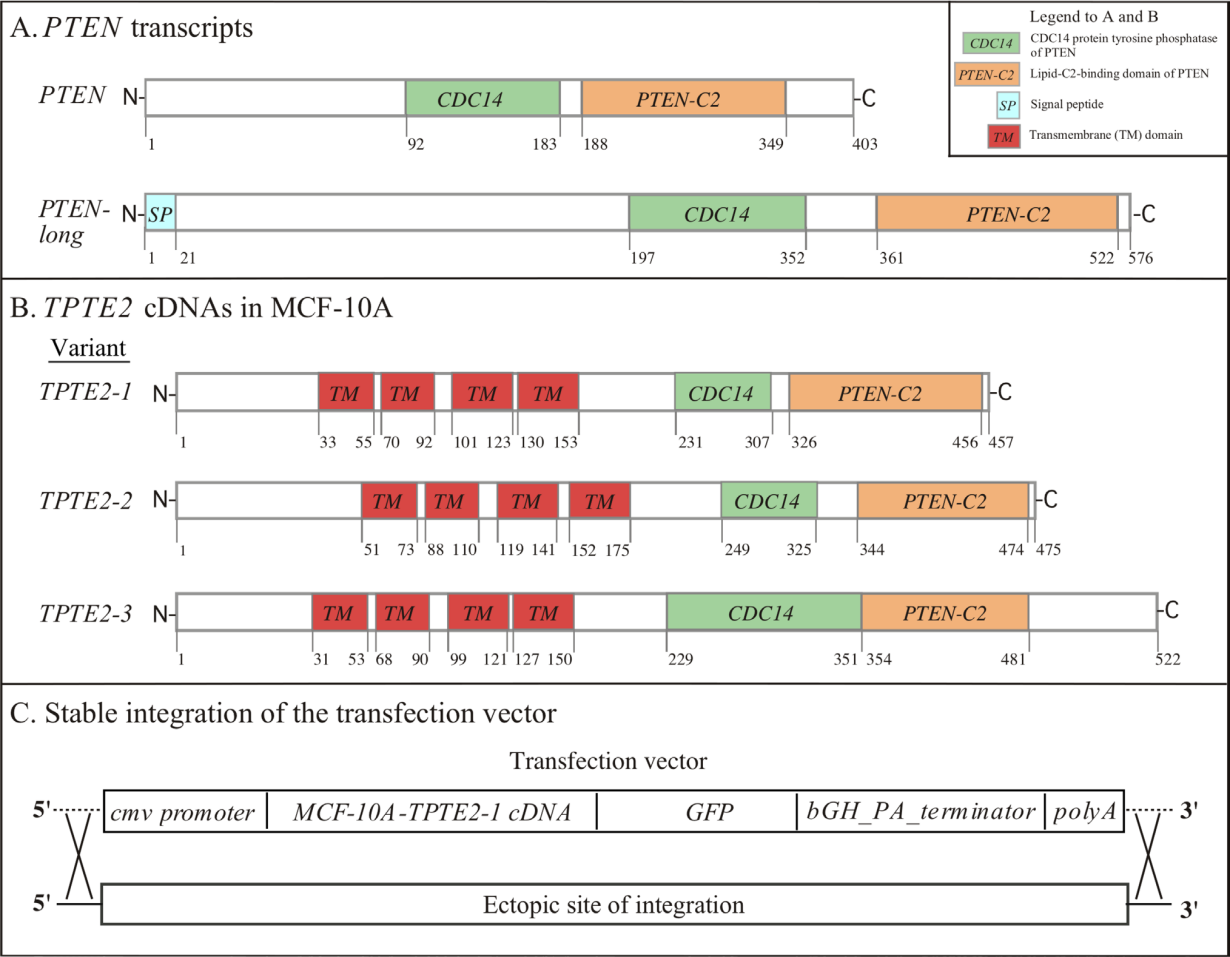
The *PTEN* and *TPTE2* transcripts, and integration of the *TPTE2* overexpression vector (**A**) The *PTEN* and *PTEN* long transcripts. (**B**) The three *TPTE2* transcript variants, *TPTE2-1, TPTE2-2* and *TPTE2-3*. (**C**) The ectopic, random integration of the construct containing the cmv promoter, *TPTE2* variant 1 (*TPTE2-1*) open reading frame, *GFP* tag, bGH-PA-terminator sequence and poly A tail. Box in upper right-hand corner of panel A shows color-coded domains in panels A and B.

The human genome contains one copy of *PTEN* [3, 5, 43], one each of the two *PTEN* homologs *TPTE1* and *TPTE2* (*TPIP*) [23, 24, 26, 27], and a pseudogene [25]. *PTEN* produces two transcripts, *PTEN* and *PTEN* long (Figure 1A) [43, 44]. Here, we have focused on the effects of overexpressing *TPTE2* (*TPIP*), which will be referred to hence as *TPTE2*, in the mutant strain *PTEN^−/−^*, the latter generated from the breast epithelial cell line MCF-10A [10]. *PTEN^−/−^* was previously shown to differ phenotypically from MCF-10A (*PTEN^+/+^*). The differences included independence of growth on growth factors, resistance to apoptosis, a decrease in susceptibility to doxorubicin and shape changes in suspension [9, 10]. TPTE2 was selected over TPTE1 (TPTE) because the former was predicted to have phosphatase activity [25, 27, 45, 46] whereas TPTE1 was predicted, based on amino acid sequence, not to have activity [26]. Amplification and sequencing of *TPTE2* from cDNA preparations of MCF-10A revealed three *TPTE2* transcripts, *TPTE2-1*, *TPTE2-2* and *TPTE2-3*, diagrammed in Figure 1B. The variants differed at both the N- and C- terminal regions (Figure 1B). All three, however, contained the CDC14 protein tyrosine phosphatase domain involved in the conversion of PIP3 to PIP2, and the PTEN-C2 domain, a lipid-binding domain involved in localization of PTEN to the inner face of the plasma membrane (Figure 1B) [3, 25]. *TPTE2-3* corresponded to *TPIP*γ, identified by Walker *et al.* [27]. All three *TPTE2* variants contained four transmembrane (TM) domains in the N-terminal half of the transcript (Figure 1B). These TMs are absent in *PTEN* (Figure 1A). *TPTE2-1* (Figure 1B) was selected for overexpression because of its high sequence homology to *PTEN*, including similar relative positions of the CDC14 and C2 domains (Figure 1A). To generate *TPTE2-1* overexpression strains in the *PTEN* mutant *PTEN^−/−^-1*, a transformation plasmid was generated that contained the *TPTE2*-*1* cDNA fused in frame to *GFP* and under the control of the cytomegalovirus (cmv) promoter (Figure 1C). The *TPTE2* overexpression plasmid integrated ectopically into the genome of the mutant strain *PTEN^−/−^-1*. Two independent clones of *TPTE2-1* overexpressors, *TPTE2^oe^-1* and *TPTE2^oe^-2*, were employed in subsequent analyses.

### Proof of overexpression

**Figure 2:**
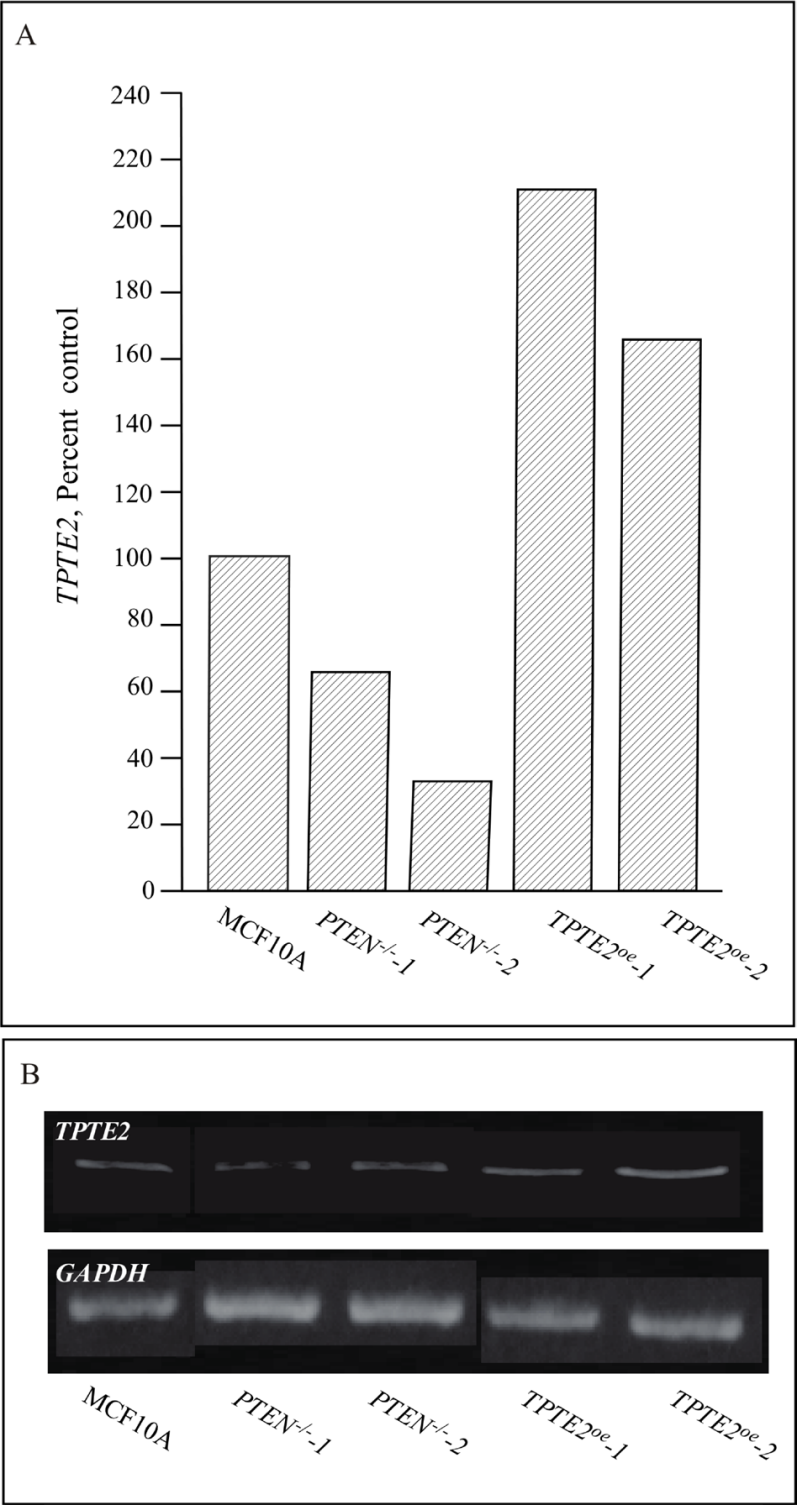
Expression of *TPTE2* in the control strain MCF-10A, the two *PTEN^−/−^* strains and the two *PTEN^−/−^* strains in which *TPTE2* is overexpressed Expression was measured by RT-PCR. (**A**) Expression of *TPTE2* as percent control (MCF-10A). (**B**) Representative gel of *TPTE2* expression. *GAPDH* expression was assessed to verify uniform loading of gels.

**Figure 3:**
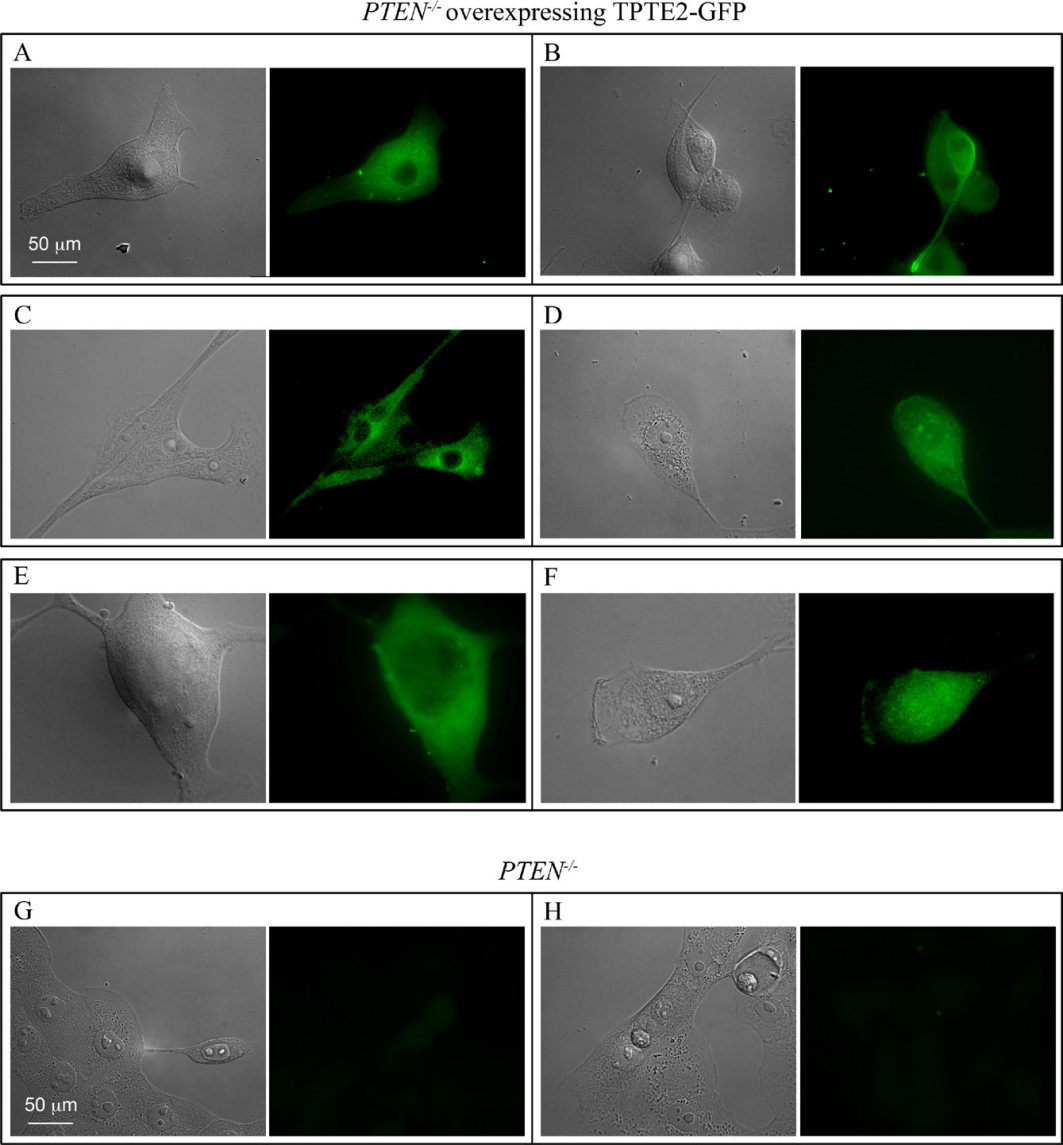
*TPTE2^oe^-1* cells express the TPTE2-GFP fusion protein (**A**–**F**) Examples of *TPTE2^oe^* cells expressing TPTE2-GFP. (**G**, **H**) Examples of *PTEN^−/−^* cells. Cells were treated with a mixture of two anti-GFP mAbs and then stained with the secondary fluorescent antibody against mouse mAb, anti H+L Alexa 488 antibody.

*TPTE2* has been found to be highly expressed in the testes, and at lower levels in spermatocytes, the brain and stomach [26, 27, 47, 48]. It is expressed at negligible levels in other tissues [47, 48]. To demonstrate that *TPTE2* was actually overexpressed in strains *TPTE2^oe^-1* and *TPTE2^oe^-2*, growth cultures were analyzed by RT-PCR. MCF-10A cells and the two *PTEN^−/−^* derivative mutants expressed *TPTE2*, but the *PTEN^−/−^* derivative mutants did so at approximately 60% and 40% that of MCF-10A cells (Figure 2A, 2B). *TPTE2-1^oe^* and *TPTE2^oe^-2*, expressed *TPTE2-1* at 3 fold and 2.5 fold, respectively the level of the *PTEN^−/−^* mutants (Figure 2A, 2B). To demonstrate that the fusion protein TPTE2-GFP was expressed, we employed indirect immunostaining, in which cells were permeabilized and fixed, treated with a mixture of two anti-GFP mAbs, DSHB-GFP-12A6 and DSHB-GFP-AC9, and then treated with the secondary fluorescent anti-mouse IgGH+L Alexa 488 mAb. Over 80% of *TPTE2-1^oe^* and *TPTE2^oe^-2* cells grown in tissue culture preparations exhibited bright diffuse staining (Figure 3A–3F), demonstrating that they expressed high levels of TPTE2-GFP throughout the cytoplasm. There was no staining in *PTEN^−/−^* mutant cells treated in a similar fashion (Figure 3G, 3H).

### Wound healing

**Figure 4:**
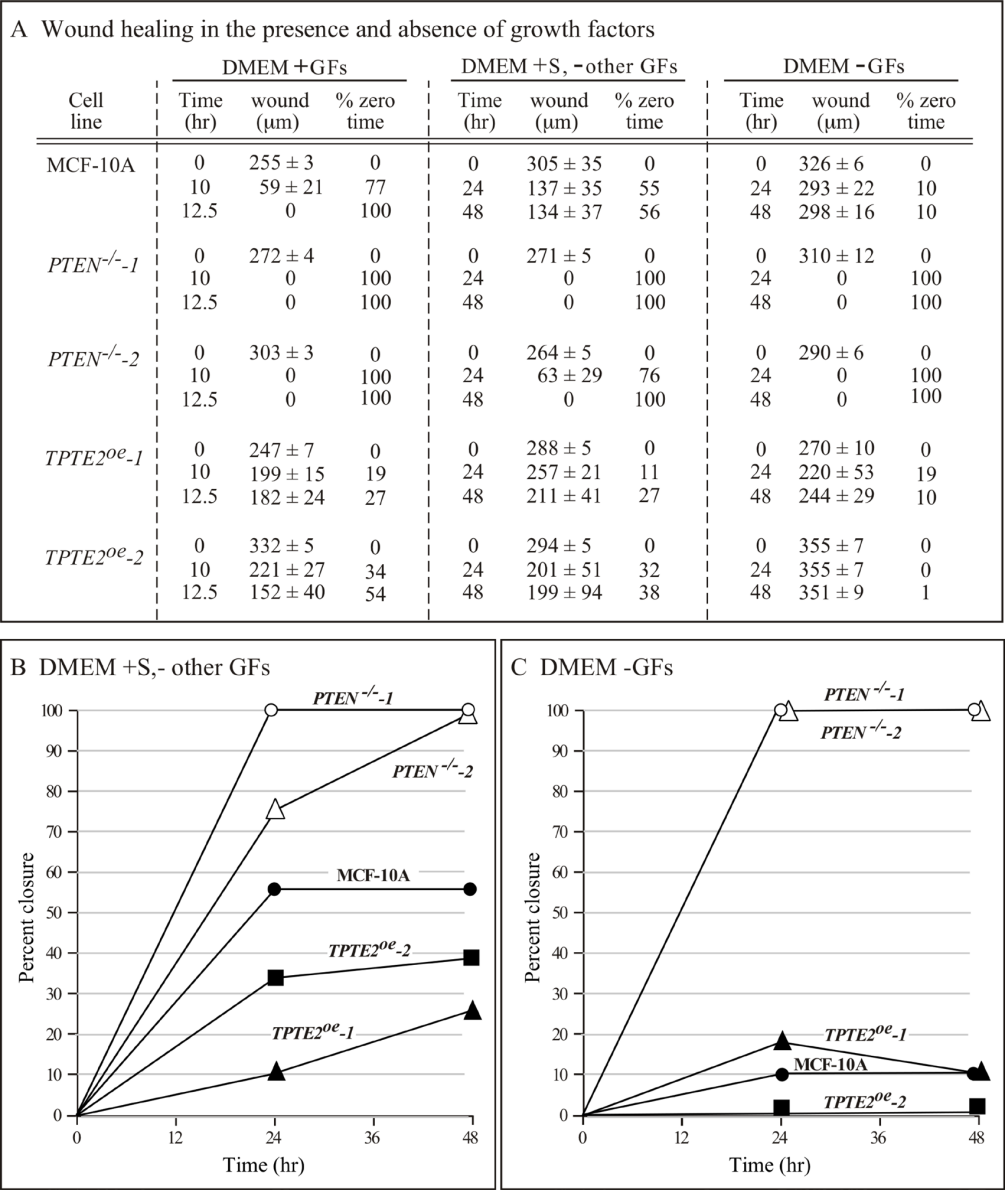
Overexpressing *TPTE2* in *PTEN^−/−^* cells rescues mutant wound healing characteristics Wound healing was tested in three media in which growth factors (GFs) were manipulated: DMEM+GFs medium, (DMEM+GFs), DMEM plus serum but lacking other GFs medium, (DMEM+S-other GFs) and, DMEM minus GFs medium (DMEM-GFs). (**A**) Wound healing data (mean ± standard deviation, *N* = 3) are presented as wound width in μm over time and percent of original wound width. Total time of experiment was predicated on the rate of total wound healing in the *PTEN^−/−^* mutant for each medium. (**B**) Data (means) graphed for wound healing as a function of time in DMEM+S, -other GFs medium. (**C**) Data graphed for wound healing as a function of time in DMEM-GFs medium.

**Figure 5:**
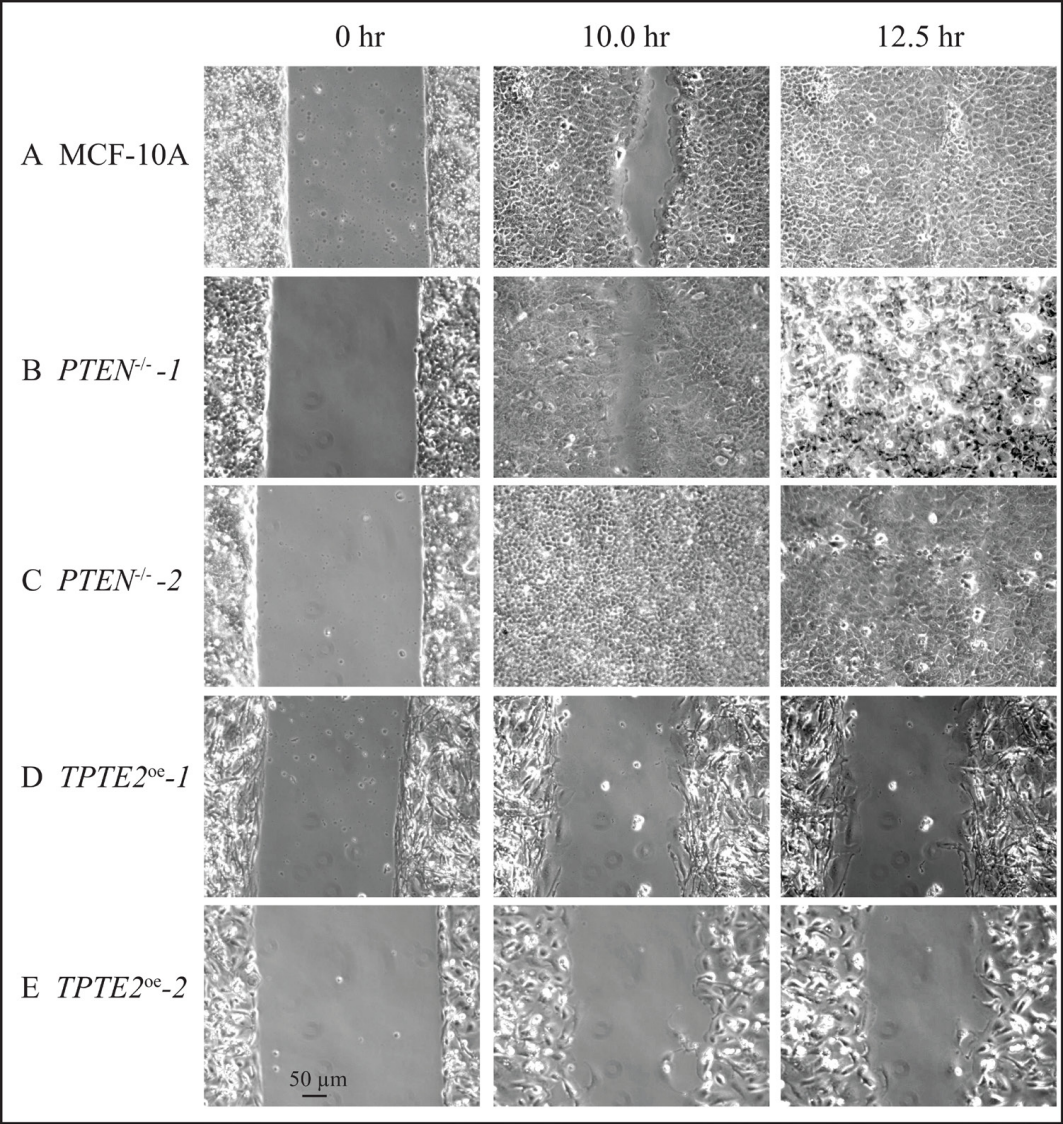
Representative images in DMEM+GFs medium reveal that the *PTEN*^−/−^ mutant undergoes wound healing faster than parental MCF-10A cells and that overexpressing *TPTE2* in *PTEN*^−/−^ cells actually retards wound healing, accentuating the parental (*PTEN*^+/+^) phenotype (**A**) MCF-10A, (**B**) *PTEN*^−/−^-1 (**C**) *PTEN*^−/−^-2, (**D**) *TPTE2^oe^*-1, (**E**) *TPTE2^oe^*-2.

In the model system *D. discoideum*, it was demonstrated that individual cells of the *ptenA^−^* (human *PTEN* ortholog) mutant exhibited a variety of motility-associated defects, including a reduction in velocity, an increase in lateral pseudopod formation and an increase in turning, which affected the efficiency of chemotaxis and thus caused defects in multicellular morphogenesis [31–37]. Overexpressing the homolog of *ptenA*, *lpten*, in the mutant *ptenA^−^* rescued all of these phenotypic defects [30]. Identification of similarly strong motility and chemotaxis differences between individual cells of strains MCF-10A and the *PTEN^−/−^* derivative strain to test whether *TPTE2* overexpression could rescue mutant defects, proved difficult because of the low velocity and weak chemotactic responses of MCF-10A and *PTEN^−/−^* cells. We therefore exploited the characteristic of collective cell migration, the basis of wound healing [49–52]. It has been suggested that collective cell migration may, in fact, more accurately reflect the motile behavior of cells during tumorigenesis and metastasis [53–55]. *PTEN* had previously been implicated in this behavior [56, 57]. We therefore tested for, and identified, strong behavioral differences between the parental MCF-10A line and the two mutants, *PTEN^−/−^-1* and *PTEN^−/−^-2* in the wound healing process. In wound healing assays, cells are either removed from a confluent monolayer by scraping a gap (“wound”) [58, 59], or by removing an insert initially positioned in a monolayer, to generate a gap (“wound”) [60]. Cells at the two edges of the opposing confluent layers in both assays then fill the gap by collective cell migration [50, 58, 59]. Leader cells move into the wound and the two opposing sheets of cells collectively move in the direction of the leader cells, filling the gap [50, 61–63]. To test wound healing, we used the latter assay [60, 64]. To assess conditions that provided the most extreme differences between parental MCF-10A cells and the two derivatives *PTEN^−/−^* strains, we grew the initial monolayer in DMEM+GFs, then monitored wound healing in the three media DMEM+GFs, DMEM+S,-other GFs and DMEM-GFs. In DMEM+GFs medium, there was an initial, small difference in the speed of wound healing between MCF-10A and the two *PTEN^−/−^* clones, observed at 10 hours (Figures 4A, 5A–5C, respectively). However, both the MCF-10A and *PTEN^−/−^* clones subsequently completed wound healing by 12.5 hours (Figures 4A, 5A–5C, respectively). Overexpressing *TPTE2* in mutant clone *PTEN^−/−^-1*, in the derivatives *TPTE2^oe^-1* and *TPTE2^oe^-2*, retarded wound healing, resulting in rates not only below that of the two *PTEN^−/−^* clones, but also below that of the parental strain MCF-10A cells (Figures 4A, 5D, 5E, respectively). Wound healing for the two *PTEN^−/−^ TPTE2^oe^* derivatives was completed in 26 hours. These results suggested that overexpressing *TPTE2-1* not only rescued the *PTEN^−/−^*-associated trait in DMEM+GFs medium, but in this case accentuated the behavioral phenotype of the parental control, MCF-10A.

**Figure 6:**
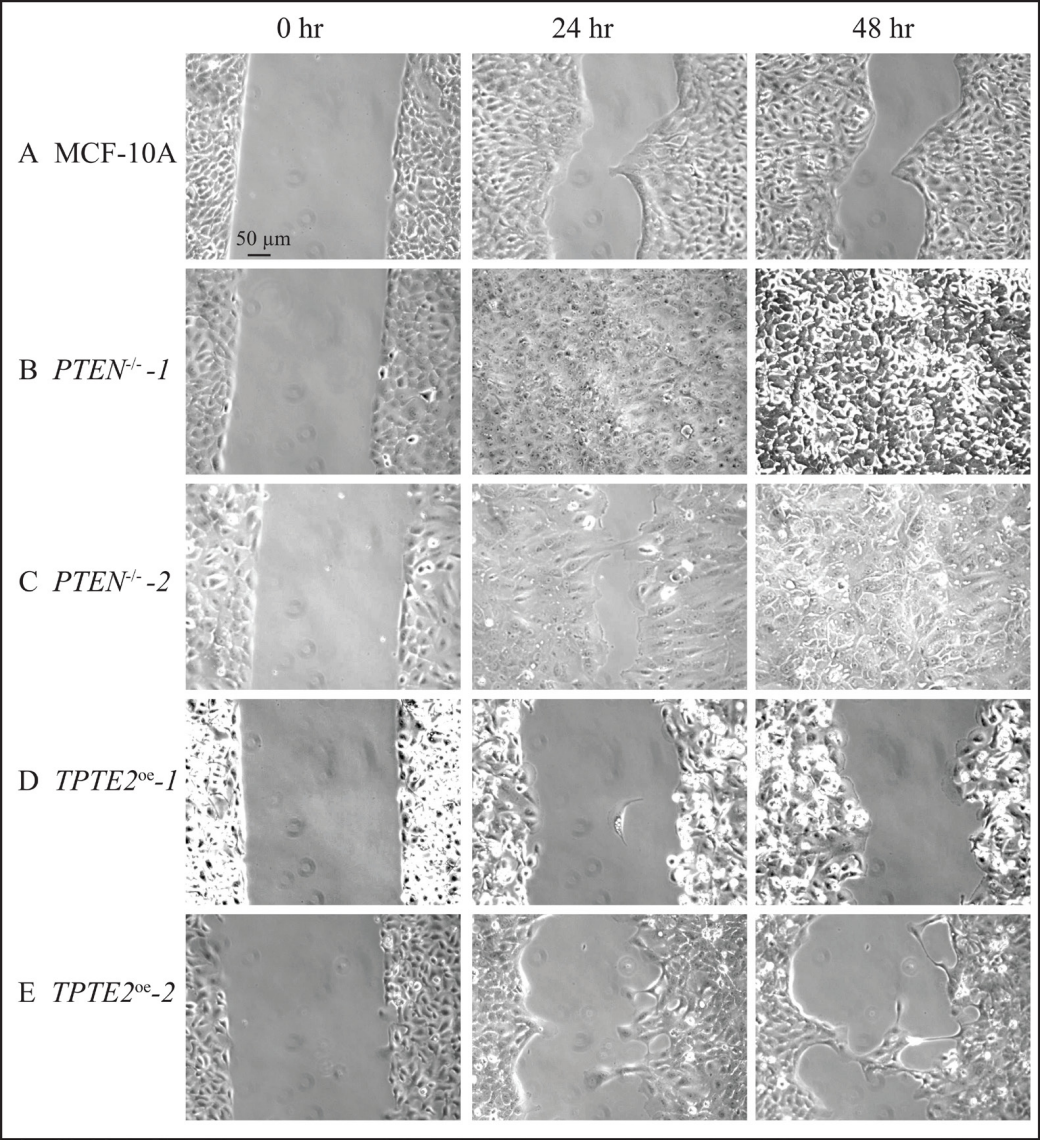
Representative images in DMEM+S, -other GFs medium reveal that overexpressing *TPTE2* in *PTEN*^−/−^ cells slows the wound healing process, thus reestablishing and actually accentuating the slower rate of the parental MCF-10A cell line (**A**) MCF-10A, (**B**) *PTEN*^−/−^*-1* (**C**) *PTEN*^−/−^*-2*, (**D**) *TPTE2^oe^-1*, (**E**) *TPTE2^oe^-2*.

When the GFs other than serum were removed, in the medium DMEM + S,-other GFs, the rate of wound healing by MCF-10A cells was dramatically reduced (Figures 4A, 4B; 6A). Parental MCF-10A cells underwent only 56% closure after 48 hours (Figures 4A, 4B, 6A). In contrast, the two *PTEN^−/−^* strains underwent either near or complete closure in 24 hours (Figures 4A, 4B, 6B, 6C, respectively). Overexpressing *TPTE2*, in *TPTE2^oe^-1* and *TPTE2^oe^-2*, reversed rapid closure of the gap and again accentuated the characteristic of slow closure exhibited by the parental line MCF-10A (Figures 4A, 4B, 6A, 6D, 6E, respectively). Whereas closure by MCF-10A was 56% complete after 48 hours, that of the two *PTEN^−/−^TPTE2^OE^* lines was 27% and 38%, respectively (Figures 4A, 4B, 6A, 6D, 6E, respectively).

**Figure 7:**
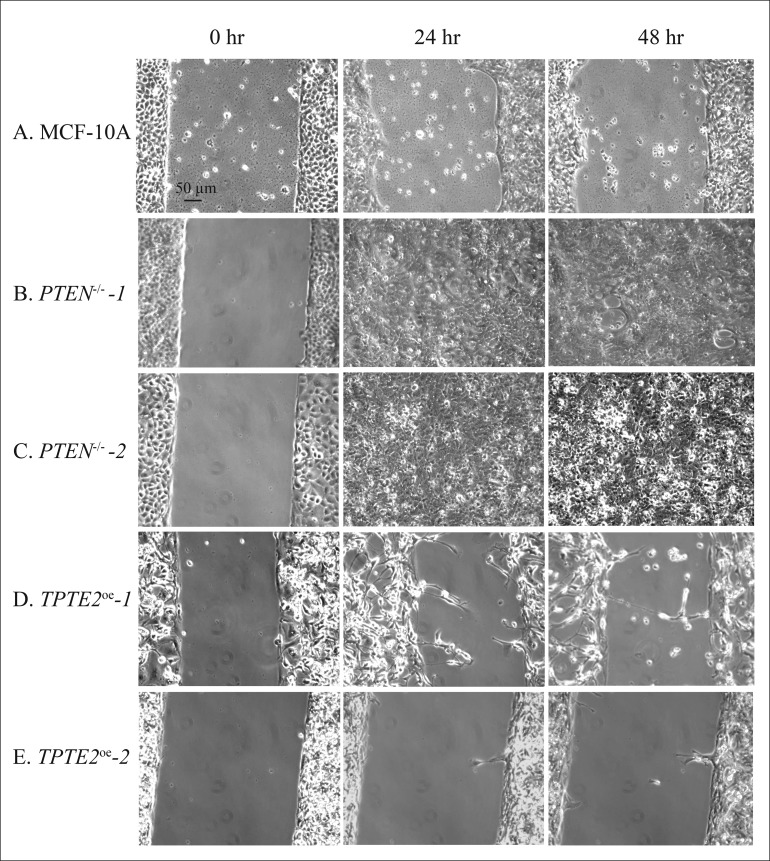
Representative images in DMEM-GFs medium reveal that overexpressing *TPTE2* in *PTEN*^−/−^ cells reverses completely GF-independent wound healing exhibited by *PTEN*^−/−^ cells, resulting in the absence of wound healing, the phenotype of parental MCF-10A cells (**A**) MCF-10A, (**B**) *PTEN*^−/−^ *-1* (**C**) *PTEN*^−/−^ *-2*, (**D**) *TPTE2^oe^ −1*, (**E**) *TPTE2^oe^ −2*

When all GFs, including serum, were omitted, in DMEM-GFs medium, MCF-10A cells underwent only 10% closure by 48 hours (Figures 4A, 7A), whereas cells of the two *PTEN^−/−^* mutants underwent 100% closure by 24 hours (Figures 4A, 4C, 7B and 7C, respectively). Overexpressing *TPTE2* in the two overexpression strains reinstated the MCF-10A phenotype (Figures 4A, 4C, 7A, 7D, 7E, respectively). The percent closure was 10 and 1%, respectively in the two *TPTE2* overexpression lines, after 48 hours (Figures 4A, 4C, 7D and 7E, respectively). Together, the results demonstrate that the slightly lower rate of wound healing by MCF-10A cells in the presence of GFs, and the extremely slow rate in the absence of GFs were lost in the *PTEN^−/−^* mutants, which undergo rapid wound healing in the presence of GFs, but reestablished in *PTEN^−/−^* mutants in which *TPTE2* was overexpressed.

### Rate of cell division in the presence of GFs

**Figure 8:**
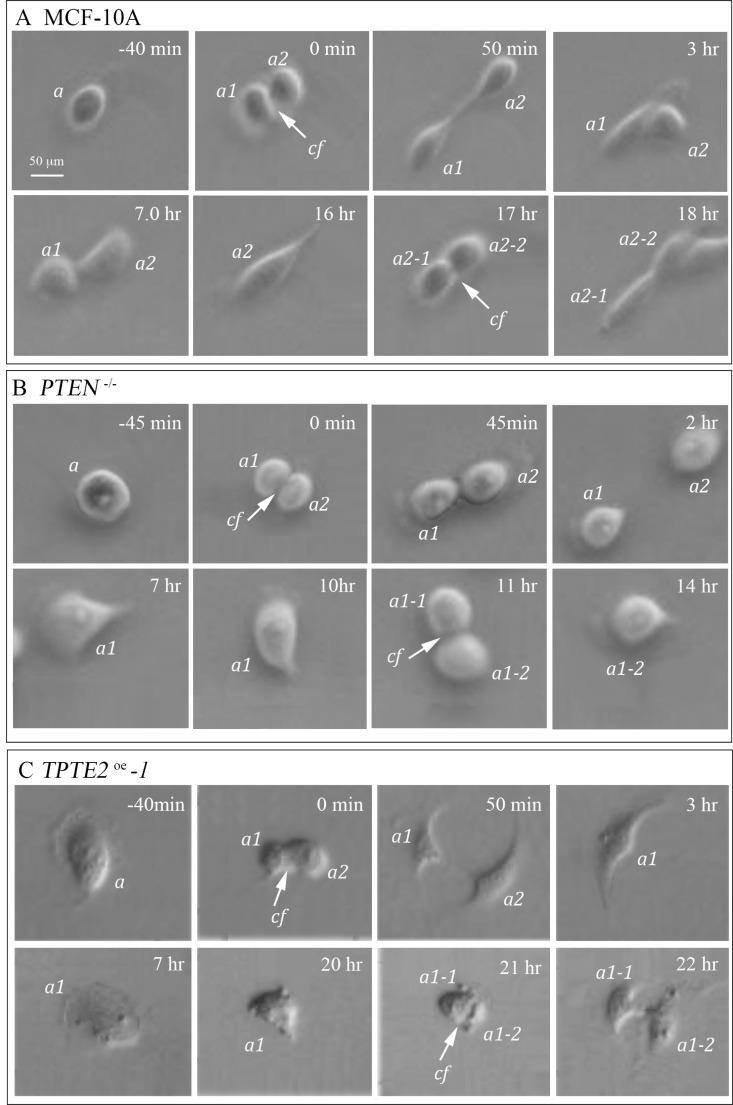
Overexpressing *TPTE2* in *PTEN^−/−^* cells slows the accelerated rate of cell division to that of parental MCF-10A cells The time between division furrows was assessed microscopically for 10 cells of MCF-10A, *PTEN^−/−^* and *TPTE2^oe^*, and found to be 17.3 ± 2.9, 11.6 ± 0.7 and 21.0 ± 4.3 hours, respectively. (**A**) Representative division of MCF-10A cells. (**B**) Representative division of a *PTEN^−/−^* cell. (**C**) Representative division of *PTEN^−/−^* cells in which *TPTE2* is overexpressed (*TPTE2^oe^-1*). a, parent cell; a1 and a2, daughter cells from first division; a1-1 and a1-2, and a2-1 and a2-2, daughter cells of second division; cf arrow, cleavage furrow.

It was previously demonstrated that loss of PTEN, in *PTEN^−/−^* mutant cells, conferred growth factor-independent proliferation [10]. We observed, both in tissue culture and in suspension cultures in DMEM+GFs medium, that it took longer for MCF-10A cells than *PTEN^−/−^* cells to reach confluence in a tissue culture dish in DMEM+GFs medium. To test whether this difference was related to a shorter cell cycle and if so, whether overexpression of *TPTE2* reinstated the slower rate of parental MCF-10A cells, we measured the time between cleavage furrows for 10 dividing cells in preconfluent preparations in DMEM+GFs medium on the plastic surface of tissue culture dishes. Examples of cleavage and the times of sequential cleavage furrows are presented for representative MCF-10A, *PTEN^−/−^* and *TPTE2^oe^-1* cells in Figure 8A–8C, respectively. No differences were evident in the actual cellular mechanics of cell division. However, the average interval times between cleavage furrows differed. The mean ± standard deviation of interval times for 10 MCF-10A cells and 10 *PTEN^−/−^-1* cells were 17.3 ± 2.9 (*N* = 10) and 11.6 ± 0.7 (*N* = 10) hours, respectively, a decrease of 33%. The difference was significant (*p* value 3 × 10^−5^). The interval time for 10 *PTEN^−/−^TPTE2^OE^ −1* cells was 21.0 ± 4.3 hours, which was not significantly different from that of MCF-10A cells (*p* value of 5 × 10^−2^), but significantly different from that of the *PTEN^−/−^-1* cells (*p* value 9 × 10^−6^). These results indicate that at the cellular level in the presence of GFs, the division rate of individual *PTEN^−/−^* cells was higher than that of MCF-10A cells and that overexpressing *TPTE2* in a *PTEN^−/−^* background returned the rate of division to that of parental MCF-10A cells.

### Growth, adhesion and viability in the absence of GFs

**Figure 9:**
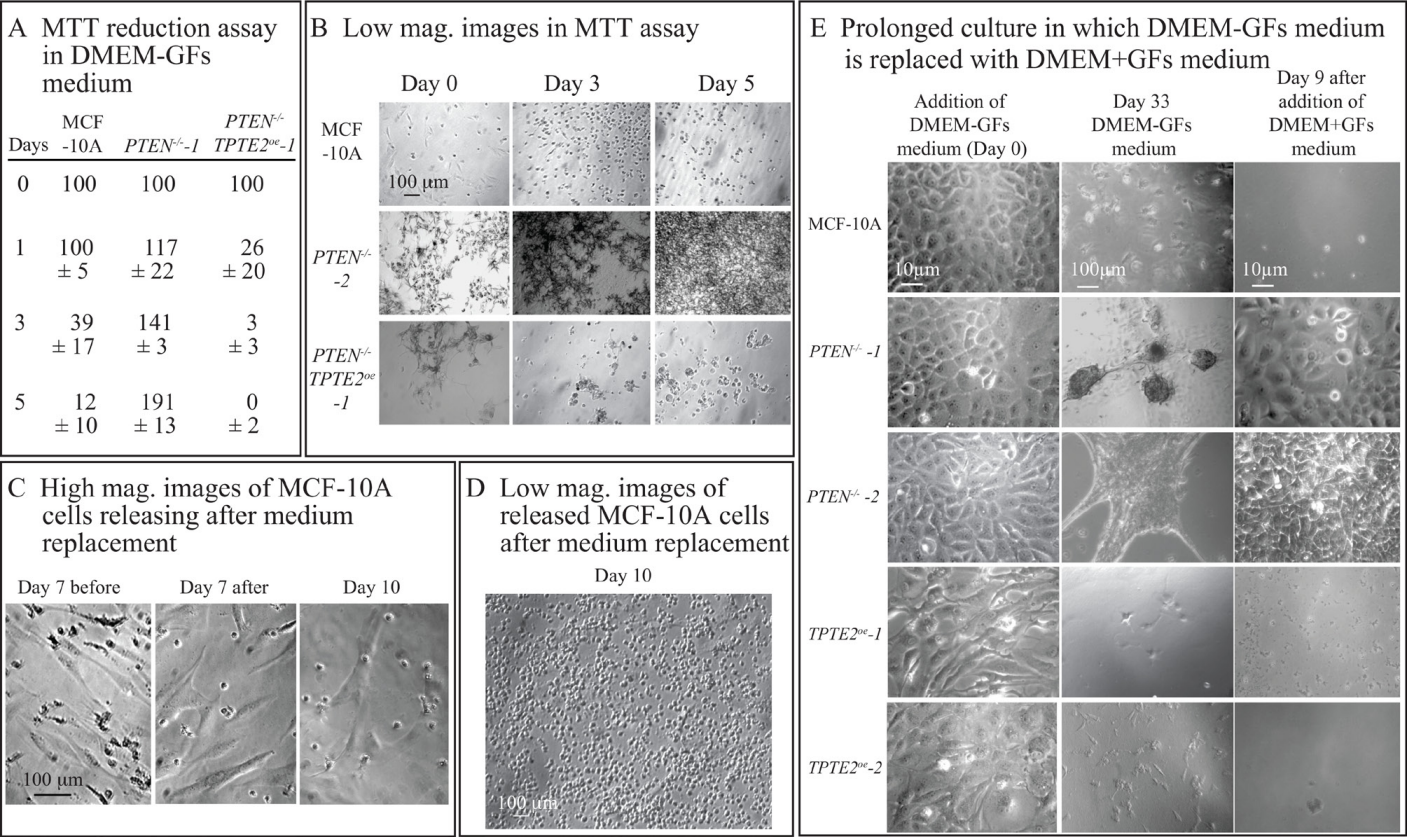
Overexpressing *TPTE2* in *PTEN^−/−^* cells reverses *PTEN^−/−^*-associated increases in viability, adhesion and growth on a 2D substrate in the absence of GFs (**A**) MTT reduction activity of undisturbed cell preparations over five days in DMEM-GFs medium. Measurements were made on total cells in the preparation. (**B**) Low magnification images of MTT preparations in panel A. (**C**) High magnification images of MCF-10A preparations on a 2D substrate, in which DMEM-GFs medium is replaced with fresh DMEM-GFs medium at seven days and incubated three additional days. Before and after medium change is noted. (**D**) Low magnification image of the supernatant of an MCF-10A cell preparation on a 2D substrate in which DMEM-GFs was replaced with fresh DMEM-GFs medium at seven days and incubated three additional days. (**E**) Images of cell preparations grown to confluency on a 2D substrate in DMEM+GFs medium (0 days), then cultured for 33 days in DMEM-GFs medium. During the 33 days, the DMEM-GFs medium was replaced every seven days with fresh DMEM-GFs. At 33 days the medium was replaced with DMEM+GFs and incubated nine additional days.

It was previously demonstrated by measuring MTT reduction in cell preparations in the wells of tissue culture plates, that the viability of MCF-10A cells was dependent upon GFs, but the viability of mutant *PTEN^−/−^* cells was independent [10]. We first analyzed whether overexpression of *TPTE2* reversed this mutant-associated characteristic, using the MTT reduction assay. In the assay MCF-10A, *PTEN^−/−^* and *PTEN^−/−^TPTE2^OE^* cells were plated in the wells of a 96 well tissue culture dish containing DMEM-GFs medium. Cultures were assayed at zero, one, three and five days for MTT reduction. MCF-10A cells lost approximately 90% of MTT reduction activity after the first day (Figure 9A). In *PTEN^−/−^* cultures, MTT reduction activity increased continuously over the five days of incubation, almost doubling by five days (Figure 9A). In *PTEN^−/−^TPTE2^OE^* cultures, MTT reductase activity was reduced by approximately 75% in one day, and by 100% in five days (Figure 9A), suggesting a loss in metabolic activity at a rate higher than in MCF-10A cultures (Figure 9A). Low magnification images of cells at the substratum of undisturbed preparations suggested that cells of all three cell lines adhered to the dish bottom for five days in DMEM-GFs, but that the density was higher in *PTEN^−/−^* cultures (Figure 9B). In a longer analysis of MCF-10A cell cultures in DMEM-GFs medium, imaged at high magnification on the substratum, we found that spread cells were still present on the substratum, but three days after changing the medium with fresh DMEM-GF medium, cell density on the substratum decreased (Figure 9C). Low magnification images of the medium revealed that cells had released into the medium and had rounded-up (Figure 9D). Similar results were obtained for *PTEN^−/−^TPTE2^OE^* cells, but few released in *PTEN^−/−^* cultures. These results together demonstrate that MCF-10A cells lose viability and release from the substratum when deprived of growth factors, but *PTEN^−/−^* derivatives are growth factors-independent, retain viability, continue to grow and remain attached to the substratum. Overexpression of *TPTE2* in *PTEN^−/−^* cells reverse GF-independence, returning the phenotype to that of MCF-10A cells and actually speeding up the loss of metabolic activity.

In a final experiment to demonstrate that long term viability acquired by *PTEN^−/−^* cells is reversed by overexpressing *TPTE2*, cells were grown to confluency in DMEM+GFs medium, and the medium then substituted with DMEM-GFs medium and cultured for 33 additional days. The DMEM-GFs medium was replaced every seven days. At the end of this period, there were very few cells on the substratum of MCF-10A cultures, whereas *PTEN^−/−^* cultures contained cell that had formed aggregates (Figure 9E). Reintroduction of GFs after 33 days resulted in essentially no growth in the MCF-10A cultures, which were visually devoid of cells, but caused the cells in the *PTEN^−/−^* aggregates to spread and grow as polylayers (Figure 9E). Overexpression of *TPTE2* and *PTEN^−/−^* cells reestablished the scenario observed for MCF-10A cells (Figure 9E).

### Viability in a 3D Matrigel matrix

**Figure 10:**
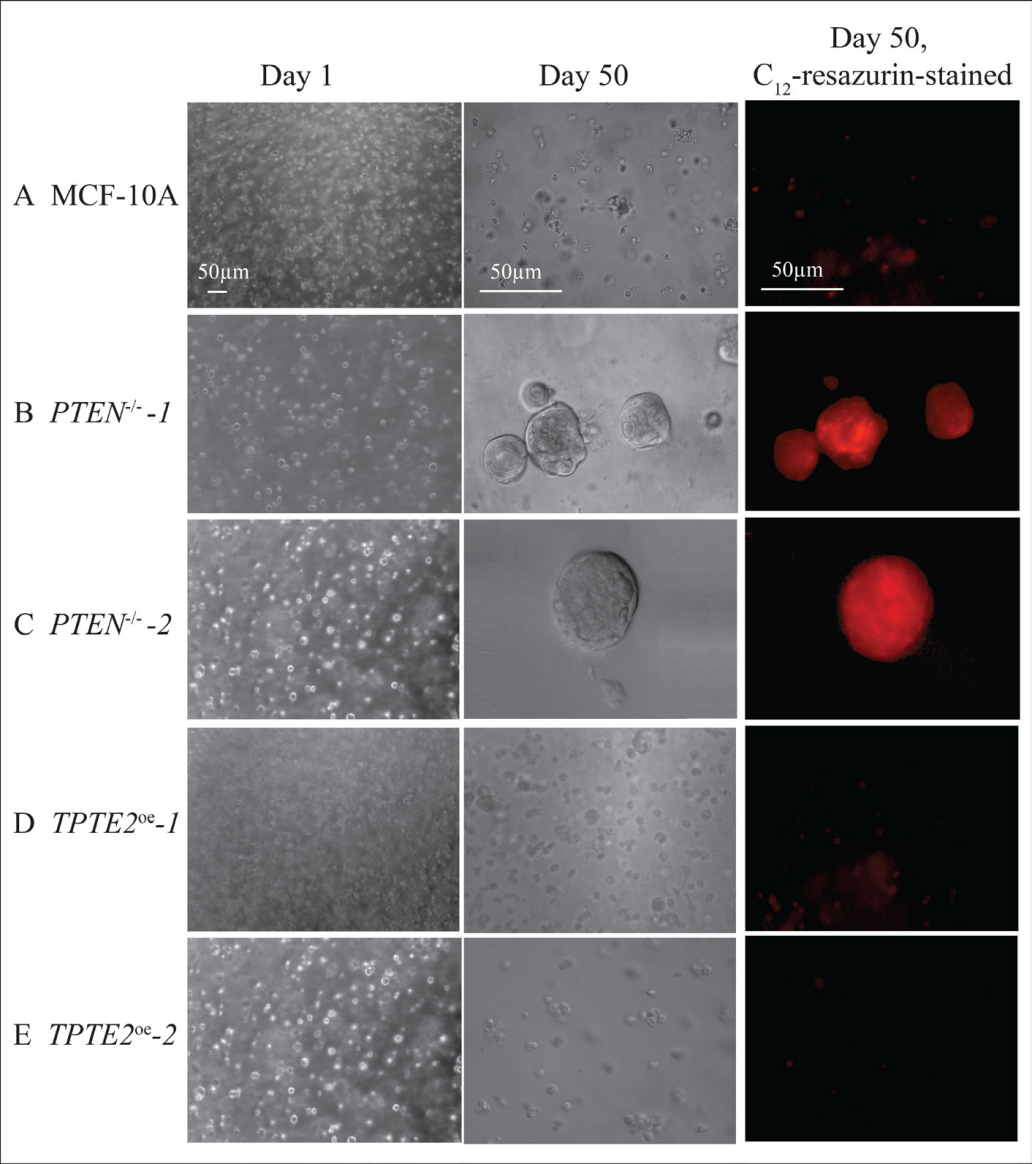
Overexpression of *TPTE2* in the *PTEN^−/−^* mutant reverses the mutant-acquired capacity of cells to form aggregates and survive for extended periods in the absence of anchorage in a 3D Matrigel model, in DMEM-GFs medium Cells were seeded in transparent 3D Matrigel in DMEM-GFs medium and incubated for 50 days, then analyzed by light microscopy and by staining with C_12_-resazurin, which identifies metabolically active cells.

Transformed cells have been demonstrated to acquire anchorage-independent growth in soft agar [65]. Previous experiments in soft agar suggested that deletion of *PTEN* in MCF-10A cells did not confer this characteristic [10]. Here, however, using a 3D model [66, 67] of transparent Matrigel, which is composed primarily of laminin, collagen and heparin sulfate proteoglycans [68], in DMEM-GF medium, we found that *PTEN^−/−^* cells acquired anchorage independent growth and viability. When seeded in 3D Matrigel and incubated for 50 days, the great majority of MCF-10A cells died off (Figure 10A). The dramatic decrease in cells was verified by the negligible levels of staining with C_12_-resazurin (Figure 10A). C_12_-resazurin identifies metabolically active cells [69]. In marked contrast the two *PTEN^−/−^* strains formed large aggregates after 50 days, which stained with C_12_ resazurin (Figure 10B, 10C). Overexpression of *TPTE2* reinstated the characteristics of cell death (Figure 10D, 10E). Similar results were obtained for 10 regions of duplicate preparations for each tested strain.

### Apoptosis marker

**Figure 11:**
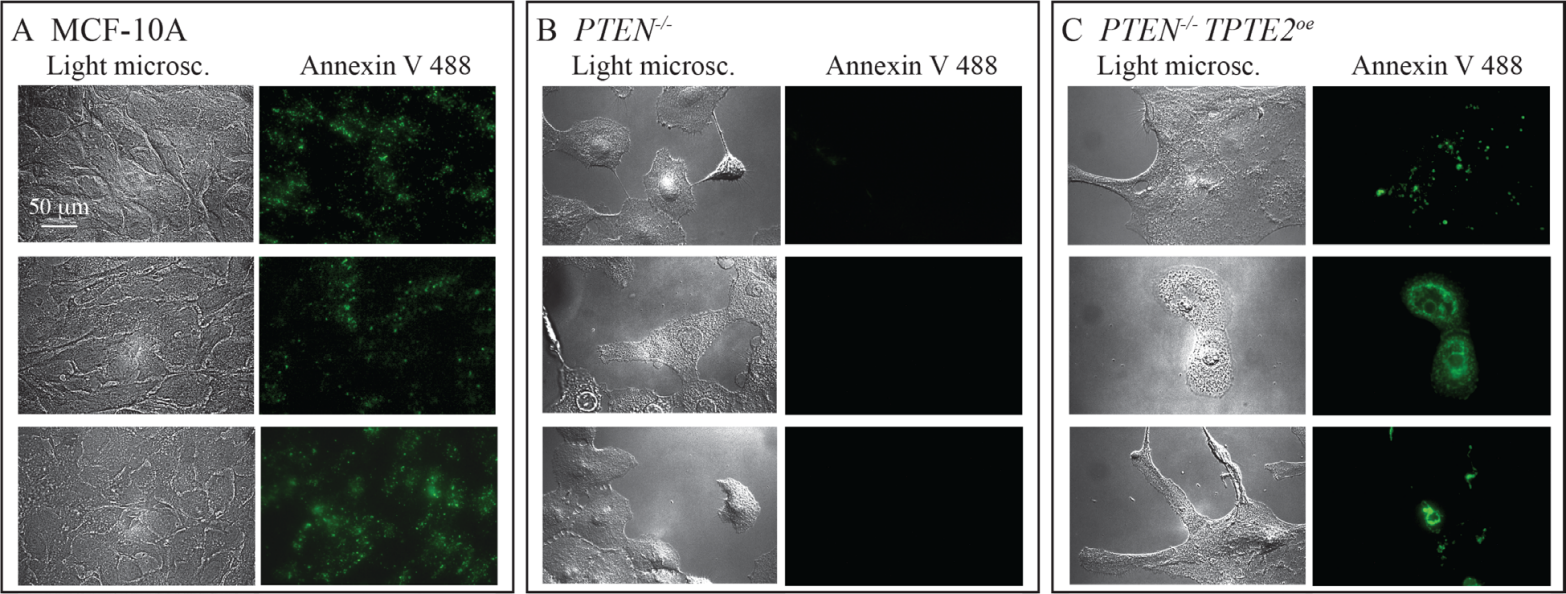
Overexpression of TPTE2 in the *PTEN^−/−^* mutant reverses the mutant-acquired loss of annexin V binding, the latter a characteristic associated with apoptosis Three pairs of representative images, light microscopic and fluorescent, are provided for annexin V-stained preparations. (**A**) MCF-10A cells. (**B**) *PTEN^−/−^* cells. *PTEN^−/−^ TPTE2^oe^*-1 cells.

Previous studies [10] and those described here demonstrated that deletion of *PTEN* results in the loss of the dependency of growth and viability on GFs and anchorage to a substratum, characteristics associated with apoptosis [38, 39]. Overexpressing *TPTE2* in *PTEN^−/−^* cells reestablished the parental charcteristics. We therefore tested whether MCF-10A cells and *PTEN^−/−^* cells overexpressing *TPTE2* expressed apoptosis-associated annexin V binding sites and whether these sites were absent in *PTEN^−/−^* cells. This was indeed the case. The majority of MCF-10A cells grown to subconfluence in DMEM+GFs stained with fluorescently conjugated annexin V (Figure 11A). *PTEN^−/−^* cells did not bind annexin (Figure 11B). When *TPTE2* was overexpressed in *PTEN^−/−^*, the majority again stained with annexin V (Figure 11C). Over 100 cells were analyzed for each cell line.

## DISCUSSION

PTEN is a phosphoinositide 3 (PIP3) phosphatase that regulates the level of PIP3 by counteracting the phosphorylation of PIP2 to PIP3 by the kinase PI3K [70]. PIP3 is a cytoplasmic signal, which functions primarily by activating the AKT pathway, which regulates membrane trafficking, membrane-cytoskeletal interactions and a number of other basic cellular processes [71–73], some of which are involved in tumorigenesis [74–76]. By maintaining a low level of PIP3, PTEN suppresses motility [6, 8, 30, 36, 77, 78], cell multiplication [4, 10, 12, 15], adhesion [10], aggregation [30, 36], and resistance to apoptosis [4, 10, 18]. Mutations in *PTEN* are found in a variety of cancers, and is considered a secondary mutation involved in metastasis and tumorigenesis [3, 5, 7, 79–81]. PTEN localizes to the plasma membrane, endoplasmic reticulum, and mitochondrial membrane [82–85]. It attaches to the plasma membrane through interactions with the CBR3 loop associated with the PTEN-C2 binding domain [85–87]. Adhesion appears to be mediated by electrostatic interactions [88]. Reversing the effects of a PTEN mutation, therefore, could suppress tumorigenesis, either inhibiting or retarding it.

One direct approach to reversing the loss of PTEN function would be to replace the mutated gene with a normal copy. In the application of this general approach to ovarian cancer involving a mutated *P53* gene, the normal *P53* gene, regulated by a strong constitutive promoter in the adenovirus vector SCH58500, was used to infect mutated cells in order to reestablish P53 function. Using this general technique in clinical trials, there were indications of increased patient survival [89–91], but the general approach has not matured to a level that leads to reliable inhibition or elimination of tumors [92–95]. Recently, we considered an alternative approach. To reestablish normal function in a *PTEN* mutant, we hypothesized that upregulating a functional homolog of *PTEN*, presumably by identifying a soluble signal or monoclonal antibody that accomplishes this, might reestablish PTEN function [30]. If this approach has any validity, it would first have to be demonstrated that a homolog of the mutated gene, when up-regulated or overexpressed, reversed the alterations in phenotype caused by the loss of *PTEN* function in a mutated cell. Our initial attempt to explore this hypothesis was performed in the model system *D. discoideum* [29]. *D. discoideum* contains *ptenA*, an ortholog of human *PTEN*, and *lpten*, a homolog of *ptenA* [31, 37]. We found that overexpressing *lpten* rescued all of the behavioral defects associated with deletion of *ptenA*, in the mutant *ptenA^−^* [30]. The defects reversed by overexpressing *lpten* in the *ptenA^−^* mutant included a decrease in persistence of motility and increased turning, an increase in lateral pseudopod formation, decreased chemotactic efficiency, and an inability to undergo normal multicellular morphogenesis [30].

Given these results in *D. discoideum*, we have here applied the strategy to test whether overexpressing a homolog of *PTEN* in a human *PTEN^−/−^* human cell line, generated by targeted mutagenesis [10], would rescue the behavioral changes caused by the mutation. In past studies, it was demonstrated that the mutant *PTEN^−/−^* cell lines differed from the parent cell line by exhibiting growth factor-independent growth, increased adherence to tissue culture plastic, and increased resistance to apoptosis in the absence of growth factors [10]. It was also demonstrated that *PTEN^−/−^* cells abnormally remodeled the cortical actin cytoskeleton to form microtentacles that adhered to a coated tissue culture dish substrate [9], a change akin to the increase in the frequency of lateral pseudopod formation by *D. discoideum ptenA^−^* cells. To test our hypothesis in the human *PTEN^−/−^* cell line *PTEN^−/−^-1*, a homolog of *PTEN*, *TPTE2* was overexpressed in *PTEN^−/−^-1*, generating two individual *TPTE2* overexpressor cell lines, *TPTE2^oe^-1* and *TPTE2^oe^-2*. We hypothesized that because TPTE2 harbored the conserved catalytic domain, CDC14, of PTEN, it had the potential to function in a similar fashion to mediate the dephosphorylation of PIP3 to PIP2, and because it contained the conserved membrane binding domain C2, it had the potential to bind to PTEN binding sites. We further hypothesized that overexpression of TPTE2 would overwhelm the TPTE2 binding sites, and excess TPTE2 would then bind to unoccupied PTEN binding sites in the *PTEN^−/−^* mutant, even if the avidity was lower. To this end, we first identified phenotypic characteristics of the *PTEN^−/−^* mutant that differed markedly from the parental strain MCF-10A. The *PTEN^−/−^* characteristics we selected included 1) accelerated wound healing in the presence of GFs; 2) independence of wound healing on GFs; 3) a decrease in the rate of cytokinesis in the presence of GFs; 4) adhesion and viability in the absence of GFs; 5) anchorage-independent growth in the absence of GFs; and 7) loss of annexin V binding sites, a characteristic associated with apoptosis (Table 1). We demonstrate that overexpression of *TPTE2* in *PTEN^−/−^* cells, in the cell lines *TPTE2^oe^-1* and *TPTE2^oe^-2*, reversed all of the changes associated with the *PTEN^−/−^* mutation and for at least one case actually accentuated the normal characteristic expressed in parental MCF-10A cells (Table 1).

**Table 1:** The mutant-acquired *PTEN^−/−^* characteristics reversed by overexpression *TPTE2*

Characteristic	Relative level^b^		
	MCF-10A	*PTEN^−/−^*	*PTEN^−/−^ TPTE2^oe^*
Wound healing, +GFs^a^	+++	++++	++
Wound healing, +S, –other GFs^a^	++	++++	+
Wound healing, –GFs^a^ Division rate, +GFs^a^	– ++	++++ ++++	– ++
Viability, on 2D substrate, –GFs^a^, undisturbed	–	++++	–
Adhesion on 2D substrate and long term viability, –GFs^a^	–	++++	–
Viability in 3D matrix, –GFs^a^	–	++++	–
Expression of annexin V surface binding site, +GFs^a^	+++	–	++++

^a^ +GFs: plus growth factors. +S, –other GFs: plus serum, minus growth factors.

^b^ “Relative level” represents a comparison to the maximum measured amongst the three genotypes. Maximum is ++++, descending (+++, ++, +) to zero (−).

### Mechanistic model

**Figure 12:**
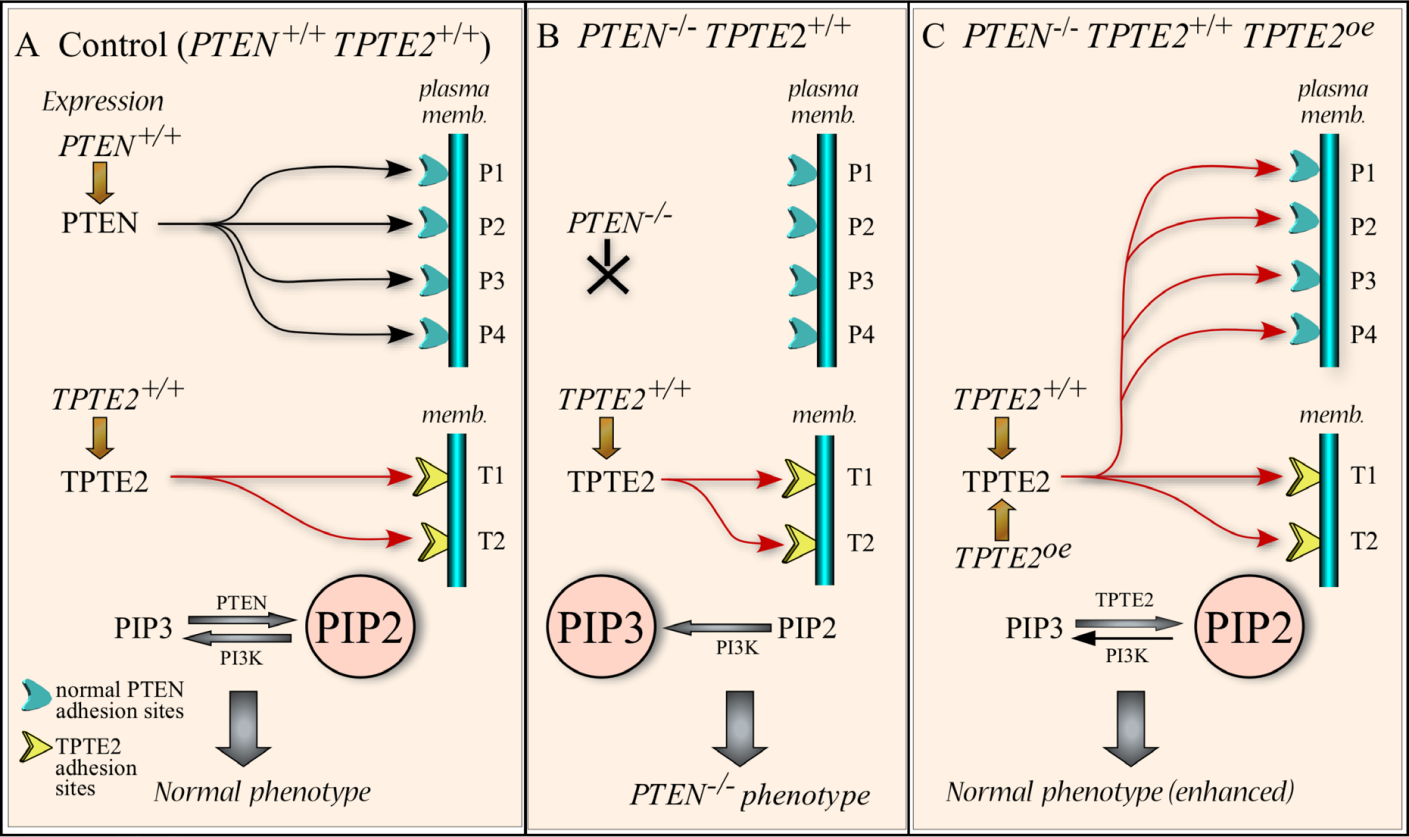
Working model for the rescue of the *PTEN^−/−^* mutant phenotype by overexpressing *TPTE2* (**A**) Model of PTEN and TPTE2 binding in control cells (*PTEN^+/+^TPTE2^+/+^*). PIP2 dominates because of PTEN dephosphorylation of PIP3. In this model of control cells, all PTEN binding sites are filled by PTEN and all TPTE2 sites by TPTE2. (**B**) Model of binding in *PTEN^−/−^* cells (*PTEN^−/−^TPTE2^+/+^*). PTEN binding sites are not occupied and PIP3 dominates because of the absence of PTEN. PTEN receptors are unoccupied whereas TPTE2 receptors are occupied by TPTE2. (**C**) Model of binding in the *TPTE2* overexpressor (*PTEN^−/−^TPTE2^+/+^TPTE2^oe^*). Overexpressed *TPTE2* leads to TPTE2 binding to both PTEN binding sites as well as TPTE2 binding sites. PIP2 dominates, resulting in a phenotype equivalent to that of control cells. P1, P2, P3, P4: PTEN binding sites. T1, T2: TPTE2 binding sites. PI3K, PI3 kinase.

Based upon the conserved domains in the PTEN homologs TPTE2, a mechanistic model of how *TPTE2* overexpression compensates for the loss of PTEN function, is developed in Figure 12. In parental cells, PTEN (black arrows) occupies membrane binding sites (blue cups) through interactions mediated by the PTEN-C2 domain (Figure 12A). At these sites, PTEN catalyzes the dephosphorylation of PIP3 to PIP2, which keeps in check the concentration of PIP3, thus suppressing PIP3-activated pathways, most notably the AKT pathway [4, 10, 96, 97], resulting in the “normal” (control) phenotype (Figure 12A). *TPTE2* (red arrows), which we have shown here is expressed in parental MCF-10A cells, binds to TPTE2 membrane receptors (yellow cups) in the endoplasmic reticulum [27] (Figure 12A). The selective binding of TPTE2 to these receptors is presumably facilitated in part by the four transmembrane domains (TMs) not present in PTEN (Figure 1), and, possibly, by the PTEN-C2 domain as well. In the *PTEN^−/−^* mutant, PIP3 levels increase due to the absence of PTEN dephosphorylation, a result of the absence of PTEN binding to PTEN sites (Figure 12B). Increased PIP3 lends to activation of the AKT pathway that results in the mutant phenotype (Figure 12B). We have shown that *TPTE2* is expressed in *PTEN^−/−^* cells, but at levels approximately half that of parental cells. We presume that in *PTEN^−/−^* cells, TPTE2 would still target its normal binding sites and, in so doing, not replace PTEN at PTEN binding sites (Figure 12B). We assume that the avidity of TPTE2 to its own binding sites would be higher than its avidity to PTEN binding sites. However, when TPTE2 is overexpressed in a *PTEN^−/−^* background, TPTE2 saturates TPTE2 binding sites, and excess TPTE2 then binds to the unoccupied PTEN binding sites (Figure 12C). It is assumed that at the PTEN binding sites, TPTE2 substitutes functionally for PTEN, resulting in a reduction in the level of PIP3, thus reinstating the control cell phenotype (Figure 12C). There are of course alternative explanations for the results obtained. For instance, the 50% reduction in *TPTE2* expression caused by deletion of *PTEN* may be basic to the *PTEN^−/−^* phenotype (i.e., the decrease in TPTE2 function results in the *PTEN^−/−^* phenotype), and overexpression of *TPTE2* rescues the phenotype simply by restoring normal TPTE2 function, not by substituting for PTEN. This alternative explanation seems less plausible since it suggests that a 50% reduction in *TPTE2* expression results in the *PTEN^−/−^* phenotype. Unfortunately, there have been no reports of a *TPTE2^−/−^* null mutant generated by targeted mutation. Our attempts to delete *TPTE2* by targeted mutation have so far failed, possibly either as a result of the high level of redundancy, which includes two *TPTE* genes, *TPTE1* and *TPTE2*, one *TPTE1* pseudogene, and seven *TPTE2* pseudogenes [23–28] (www.ncbi.nim.nih.gov/gene; / www.uniprot.orgQ6xP23.www.ensemble.org), or the fact that the *TPTE2* genes map to C-heterochromatin regions [98], which are less accessible to integration and, therefore, less amenable to targeted mutagenesis [99, 100].

## MATERIALS AND METHODS

### Growth and maintenance of cell lines

Two independent *PTEN^−/−^* mutants were previously generated by targeted mutagenesis, clone 1 (*PTEN^−/−^-1*) and clone 2 (*PTEN^−/−^-2*). Both contained targeted disruption of exon 2 of both *PTEN* alleles [10]. The parental strain of the mutants, MCF-10A, a non-tumorigenic human cell line derived from normal breast epithelium [101], the two derivative *PTEN^−/−^* strains and the two *TPTE2*-overexpressing lines generated here from *PTEN^−/−^-1* (*TPTE2^OE^ −1*, and *TPTE2^OE^ −2*), were cultured in DMEM/F12 (DMEM) medium (Life Technologies, Carlsbad, CA, USA) supplemented with horse serum (S), human recombinant EGF (EGF), insulin (I), hydrocortisone (HC) and cholera toxin (CT), all obtained from Sigma Aldrich (St. Louis, MO, USA), and a penicillin- streptomycin mixture, obtained from Thermo-Fischer (Grand Island, NY) [10]. This fully supplemented medium will be referred to as “DMEM+ GFs medium”, “GFs” referring to the full complement of growth factors. Medium containing serum, but lacking the other GFs (otherGFs: E, I, HC, CT) will be referred to as “DMEM+S,-otherGFs medium”, and medium lacking all growth factors will be referred to as “DMEM-GFs medium”. In the case of “DMEM+S,-otherGFs medium”, growth factors were charcoal-stripped (Valley Biomedical, Winchester, VA) from serum prior to its addition to DMEM medium.

### Generating *PTEN^−/−^TPTE2^OE^* strains

RNA of the parental strain MCF-10A was isolated as previously described in detail [30, 102], using Trizol (Life Technologies, Carlsbad, CA, USA) according to the manufacturer's protocol. A 3ʹ RACE system with reverse transcriptase (Life Technologies, Carlsbad, CA, USA) was used to generate cDNA using 4 μg of total RNA and the primer AUP. The three identified *TPTE2* variant cDNAs (*TPTE2* variant 1 accession number KX610659, *TPTE2* variant 2 accession number KX752070 and *TPTE2* variant 3 accession number NM_199254) were amplified from the cDNA template using primers specific for the 5ʹ and 3ʹ ends of the *TPTE2* transcript. Primers were designed using information provided by the EMBL (http://www.ensembl.org) for the *TPTE2-1* variant. cDNA was amplified using the primers *TPTE2-1* FW (5ʹ -TCCACCCACCCATGAATTATCAGGGAGTGAACCCAGAGGCACGTATGA ATGAAAGTCC-3ʹ) and *TPTE2-1* RT (5ʹ -ATTTTCTCGCCAAAAAGTATCTCCACAGCAAATTCTGG-3ʹ). PCR was performed using a Long-Range Polymerase (Roche, Indianapolis, IA, USA) /Taq-Polymerase (NEB) mixture, and a regime that included 5 min denaturation at 95° C and 40 cycles, each consisting of 20 seconds at 92° C, 30 seconds at 47° C and 4 min at 70° C. This was followed by a final elongation step of 10 min at 72° C. Fragments were gel extracted (Qiagen, Ventura, CA, USA) and cloned into the plasmid pCR4.0 (LifeScience, Carlsbad, CA) for sequencing. Sequencing was performed at the Roy J. Carver Center of Genomics in the Department of Biology of the University of Iowa (http://biology.uiowa.edu/ccg). The *TPTE2* variant *TPTE2-1* was used for insertion into the transforming vector. The 5ʹ-3ʹprimers were designed to begin at nucleotide 1383 in order to obtain inframe fusion with GFP in the plasmid pcDNA3.1/CT-GFP TOPO (Lifescience, Carlsbad, CA). The stop codon was omitted to ensure that the *TPTE2* and *GFP* sequences were inframe and produced as a fusion protein. *PTEN^−/−^* cells were grown in OptiMEM medium (Life Science, Carlsbad, CA) in 24 well tissue culture plates overnight. Fugene (Promega, Madison, WI) was employed to transfect cells with 1 μg/ml of plasmid containing the *TPTE2*-*1*-*GFP* fusion, according to the manufacturer's instructions. *PTEN^−/−^* cells were subjected to a G418 (Sigma-Aldrich, St. Louis, MO, USA) kill curve. The concentration of G418 used for selection was 150 μg/ml. Two *TPTE2^OE^* clones were selected for further analysis using G418, *TPTE2^OE^ −1* and *TPTE2^OE^ −2*. Note that both produced *TPTE2-GFP* transcripts.

### RT-PCR

RNA was prepared as described in the previous section, using 2-step LongRange RT-PCR (Qiagen, Ventura, CA, USA). For reverse transcription, an Oligo-dT primer provided by the manufacturer was employed. Amplification was performed using the Roche Expand Long Template Kit (Roche, Indianapolis, IN). Primers used to amplify a 1.135bp *TPTE2-GFP* fusion fragment were ATG120TPTE2 5ʹ-ATGGACACATTTAGTTCGACT TCTACG-3ʹstarting at position 415 of the *TPTE2-1* cDNA and *GFP* Reverse(CT-GFP) 5ʹ-GGTAAGCTTTCCGTAT GTAGC-3ʹending at position 131 of the *GFP* cDNA. To analyze expression levels by RT-PCR, RNA was isolated as described above. Reverse Transcription was performed using Quantitec RT-PCR (Qiagen, Ventura, CA, USA). One μg of total RNA was denatured at 65° C for 5 min, incubated in gDNA wipeout buffer for 2 min at 42° C and immediately placed on ice. Reverse transcription was performed for 30 min at 42° C with an Oligo-dT primer. The reaction was terminated by incubation at 95° C for 3 min. Fifty ng of cDNA was used for amplification of a 200 bp *TPTE2* fragment. The primers were RTFw 5ʹ-TGGTTTGTGCCCTCCTTATTGCC-3ʹand RTRv 5ʹ-

TCACATCATCATACAGAGGTGGACCG −3ʹ. GAPDH was used as a loading control, using the primers *GAPDH* Fw 5ʹ-CATCTTCTTTTGCGTCGCC-3ʹand *GAPDH*rv 5ʹ-CTACACTGAGCACCAGGTGGTCTC-3ʹ, to amplify an 891 base pair fragment. PCR was performed as described above, with minor changes. Primer elongation was performed for 1 min through 30 cycles for a *GAPDH* control fragment and through 40 cycles for the *TPTE2* fragment. Gel-Electrophoresis was performed using a 3% agarose gel in TBE buffer. Images were taken with UV light under subsaturating conditions. Gel band image analysis was performed using Adobe Photoshop. To measure TPTE2 expression, integrated pixel density of the band area was determined. The integrated density is the sum of the value of the pixels in a band, divided by the area of the band. TPTE2 expression was normalized to GAPDH expression determined in the same manner.

### Immunostaining TPTE2-GFP

To immunostain the fusion protein in overexpression strains, cells were washed 3 times in TBS pH7.6/0.05% Tween 20 to remove growth media. Cells were fixed in 3.8% formaldehyde (Sigma Aldrich, St.Louis, MO) for 12 min. Fixed cells were washed 3 times in tris-buffered saline (TBS) pH7.6 containing 1% BSA (ACROS, Geel, Belgium), 0.05% Tween 20, to remove growth media. Fixed cells were washed 3 times in the TBS-Tween 20 buffer. Preparations were incubated for 2 hours in TBS pH7.6/1% BSA, 0.05% Tween 20 and 0.1% Triton X-100. Cells were then washed 3 times in the TBS-Tween 20 buffer. One ml of a 1:1 mixture of the anti-GFP monoclonal antibody (mAb) DSHB-GFP-12A6 and the anti-GFP mAb DSHB-GFP-4C9 (Developmental Studies Hybridoma Bank, University of Iowa, Iowa City, IA 52245, USA [103], containing 10 μg of mAbs in TBS (pH7.6) plus 1% BSA, was added and cells incubated overnight. Excess primary antibody was removed by washing the cells 3 times with TBS (pH7.6) plus 0.05% Tween 20. The affinity-purified fluorescent anti-IgG H+L Alexa 488 antibody (Jackson ImmunoResearch, Westgrove, PA, USA) was used as a secondary antibody. It was diluted 1:300 in TBS (pH 7.6) plus 0.05% Tween 20, added and the preparation incubated for 2 hours. Cells were then washed 3 times in TBS (pH7.6) plus 0.05% Tween 20, and analyzed using a Nikon TE2000 inverted epifluorescent microsope (Nikon Instruments, Melville, NY, USA). Images were taken with a with an EOS Rebel T3i/EOS 600D camera (Canon, Lake Success, NY, USA).

### Wound healing assay

A wound healing assay was employed that consisted of a two well culture dish in which the wells were separated by an insert, which, when removed, formed a gap (“wound”) (IBIDI, Madison, WI, USA). The assay monitored collective directional migration into the gap between the opposing confluent layers of cells [60]. Experiments were performed in an incubator at 37° C in 5% CO_2_. The assay was performed according to the manufacturer's directions. In brief, 80 μl aliquots, containing 4 × 10^4^ cells were inoculated into each of the two wells and grown for 24 hours to confluency in DMEM+GFs medium. The insert was then removed, leaving a 200 μm gap between the opposing confluent layers of cells. Two ml of fresh medium (DMEM+GFs, DMEM+S,-otherGFs or DMEM-GFs) were then added. Migration into the wound was monitored microscopically through an Olympus CK2 inverted microscope equipped with a digital XCD-V50 camera (Sony, San Diego, CA, USA). Images were obtained at noted time intervals.

### Assessment of the rate of cell division

Cells at a concentration of 10^4^ per ml were inoculated into DMEM+GFs medium in a tissue culture dish and incubated in 5% CO_2_ at 37° C. Individual cells, which distributed independently on the dish bottom, were monitored through an Olympus CK2 inverted microscope equipped with a XCD-V50 digital camera (Sony, San Diego, CA, USA). Images were obtained every 45 seconds. The time of cell division was taken as the period between the formation of cleavage furrows. Ten dividing cells of each line were analyzed. Images were obtained as previously described.

### MTT reduction assay

The MTT reduction assay was performed according to procedures previously described [104, 105]. In brief, 3 × 10^3^ cells in 150 μl of DMEM-GFs medium from cultures grown to near confluency in DMEM+GFs, were plated into wells of a 96 well tissue culture plate (Midwest Scientific, Valley Park, MO, USA). After 0, 1, 3 and 5 days 20 μl of 5 mg/ml MTT, 3-(4,5-Dimethylthiazol-2-yl)-2,5-diphenyltrtrazolium bromide (Life Technologies, Carlsbad, CA, USA), were added. The plates were then incubated subsequently for 3.5 hour at 37° C in the absence of light. Microscopic images, were then taken using an Olympus CK2 inverted microscope equipped with a digital XCD-V50 camera (Sony, San Diego, CA, USA), and then150μl of a MTT solvent containing 8mM HCl, 0.2% Nonidet P-40 (NP40, Amresco, Solon, OH) in isopropanol was immediately added to each culture. The 96 well plates were then shaken for 15 min at 100 rpm protected from light at room temperature. The absorbance was determined using a Spectra max Plus 384 multiwell plate reader (Molecular Devices, Sunnyvale, CA, USA) at 590 nm. All cultures were compared to day 0. Experiments were performed in triplicate.

### Viability in the absence of growth factors on a 2D substrate

Cells were first grown in DMEM+GFs medium to confluency in the wells of a 24 well tissue culture dish. In a first approach, the DMEM+GFs medium was gently replaced with DMEM-GFs medium (zero hours), cultured undisturbed for seven days, the DMEM-GFs medium gently replaced at seven days with fresh DMEM-GFs medium and cultured three additional days. Microscopic images were taken at high magnification at seven days before and after fresh medium replacement, and at 10 days. Microscopic images were also taken of supernatant at low magnification at 10 days. In a second, long term approach, DMEM+GFs medium was replaced with DMEM-GFs, and the DMEM-GFs medium gently replaced at 9, 18 and 24 days. At 33 days, the DMEM-GFs medium was replaced with DMEM+GFs medium and incubated for nine additional days. The cell preparations were monitored microscopically with time and images obtained as described for the wound healing assay.

### Cell cultures a 3D Matrigel model

Cells grown in DMEM+GFs medium were randomly dispersal in a 3D Matrigel model in DMEM-GFs, as described previously in detail [66, 67, 106–108]. In brief, a 30 mm glass insert at the bottom of a customized culture dish was coated with Matrigel previously hydrated in DMEM-GFs for 20 min at 5° C. This preparation was then incubated at 37° C for one hour to cause gelation. Then 5 × 10^6^ cells, grown in DMEM+GFs medium, were resuspended in 250 μl of DMEM-GFs medium, added to 500 μl of (5%) hydrated GF-reduced Matrigel (Corning, Life Science, Corning, NJ) at 5° C and the cell-Matrigel mixture distributed atop the basal cushion of Matrigel and incubated at 37° C in 5% CO_2_ for 30 min, which caused Matrigel gelation. After gelation, cells were dispersed randomly throughout the 3D gel. At 50 days C_12_-Resazurin (Life Technologies, CA) was added directly to the dishes at a final concentration of 500 nM and incubated for 1 hour. Metabolically active cells reduce nonfluorescent C_12_-Resazurin to fluorescent C_12_-resorufin. Preparations were imaged with a Nikon TE2000 inverted fluorescence microscope (Nikon Instruments, Melville, NY, USA) equipped with an EOS Rebel T3i/EOS 600D camera (Canon, Lake Success, NY, USA).

### Annexin V binding

Cells were seeded on a 30mm glass botton dish (Cellvis, Mountain View, CA, USA) and grown for 2 days in DMEM+GFs. Cell death was assessed using Annexin V CF 488A conjugate (Biotium, Fremont, CA, USA) on unfixed cells according to the manufacturer's instructions. In brief, media was first removed and supernatant examined and substrate microscopically imaged. Cells on the substratum were then washed two times in 1X binding buffer (Biotium, Fremont, CA, USA). Cells were incubated in staining solution containing binding buffer and 1.25 μg/ml Annexin V conjugate for 30 min at room temperature. Stained cells were washed two times with binding buffer. Cells were imaged using a Nikon TE2000 inverted epifluorescent microsope (Nikon Instruments, Melville, NY, USA). Images were taken with an EOS Rebel T3i/EOS 600D camera (Canon, Lake Success, NY, USA) within 1 hour, as recommended by the manufacturer

## CONCLUSIONS

We have explored the hypothesis that loss of function resulting from a mutation or an epigenetic effect, of a gene that suppresses metastasis and/or tumorigenesis, may be reversed by up-regulating or overexpressing a functional homolog of that gene in the mutated cell. We previously demonstrated that this could be accomplished in a model system for a mutant of a *PTEN* ortholog [30], and here we have demonstrated it in a human epithelial cell line for a targeted *PTEN* mutant. These results support the possibility that this strategy may have the potential to be translated into a therapy for tumorigenesis. The next step in this strategy is to screen for a compound or antibody that, through interaction with a receptor, up-regulates *TPTE2* in *PTEN^−/−^* cells. This general approach may also be applicable to other cancer-associated genes with known homologs.
